# Immuno-Thrombotic Complications of COVID-19: Implications for Timing of Surgery and Anticoagulation

**DOI:** 10.3389/fsurg.2022.889999

**Published:** 2022-05-04

**Authors:** Connor M. Bunch, Ernest E. Moore, Hunter B. Moore, Matthew D. Neal, Anthony V. Thomas, Nuha Zackariya, Jonathan Zhao, Sufyan Zackariya, Toby J. Brenner, Margaret Berquist, Hallie Buckner, Grant Wiarda, Daniel Fulkerson, Wei Huff, Hau C. Kwaan, Genevieve Lankowicz, Gert J. Laubscher, Petrus J. Lourens, Etheresia Pretorius, Maritha J. Kotze, Muhammad S. Moolla, Sithembiso Sithole, Tongai G. Maponga, Douglas B. Kell, Mark D. Fox, Laura Gillespie, Rashid Z. Khan, Christiaan N. Mamczak, Robert March, Rachel Macias, Brian S. Bull, Mark M. Walsh

**Affiliations:** ^1^ Department of Emergency Medicine, Henry Ford Hospital, Detroit, MI, United States; ^2^ Department of Surgery, Ernest E. Moore Shock Trauma Center at Denver Health, Denver, CO, United States; ^3^ Pittsburgh Trauma Research Center, University of Pittsburgh Medical Center, Pittsburgh, PA, United States; ^4^ Indiana University School of Medicine South Bend Campus, Notre Dame, IN, United States; ^5^ Department of Internal Medicine, Saint Joseph Regional Medical Center, Mishawaka, IN, United States; ^6^ Department of Neurosurgery, Saint Joseph Regional Medical Center, Mishawaka, IN, United States; ^7^ Division of Hematology and Oncology, Department of Medicine, Northwestern University Feinberg School of Medicine, Chicago, IL, United States; ^8^ Mediclinic Stellenbosch, Stellenbosch, South Africa; ^9^ Department of Physiological Sciences, Stellenbosch University, Stellenbosch, South Africa; ^10^ Division of Chemical Pathology, Department of Pathology, Faculty of Medicine and Health Sciences, Stellenbosch University and National Health Laboratory Service, Tygerberg Hospital, Cape Town, South Africa; ^11^ Division of General Medicine, Department of Medicine, Faculty of Medicine and Health Sciences, Stellenbosch University and Tygerberg Hospital, Cape Town, South Africa; ^12^ Division of Medical Virology, Faculty of Medicine and Health Sciences, Stellenbosch University, Cape Town, South Africa; ^13^ Department of Biochemistry and Systems Biology, Institute of Systems, Molecular and Integrative Biology, Faculty of Health and Life Sciences, University of Liverpool, Liverpool, United Kingdom; ^14^ The Novo Nordisk Foundation Centre for Biosustainability, Technical University of Denmark, Kgs. Lyngby, Denmark; ^15^ Department of Quality Assurance and Performance Improvement, Saint Joseph Regional Medical Center, Mishawaka, IN, United States; ^16^ Department of Hematology, Michiana Hematology Oncology, Mishawaka, IN, United States; ^17^ Department of Orthopaedic Trauma, Memorial Hospital South Bend, South Bend, IN, United States; ^18^ Department of Cardiothoracic Surgery, St. Joseph Regional Medical Center, Mishawaka, IN, United States; ^19^ Department of Plastic and Reconstructive Surgery, St. Joseph Regional Medical Center, Mishawaka, IN, United States; ^20^ Department of Pathology and Human Anatomy, Loma Linda University School of Medicine, Loma Linda, CA, United States

**Keywords:** COVID-19, elective surgical procedure, immunothrombosis, obstetrics, orthopedic procedures, venous thromboembolism, thrombophilia, fibrinolysis

## Abstract

Early in the coronavirus disease 2019 (COVID-19) pandemic, global governing bodies prioritized transmissibility-based precautions and hospital capacity as the foundation for delay of elective procedures. As elective surgical volumes increased, convalescent COVID-19 patients faced increased postoperative morbidity and mortality and clinicians had limited evidence for stratifying individual risk in this population. Clear evidence now demonstrates that those recovering from COVID-19 have increased postoperative morbidity and mortality. These data—in conjunction with the recent American Society of Anesthesiologists guidelines—offer the evidence necessary to expand the early pandemic guidelines and guide the surgeon’s preoperative risk assessment. Here, we argue elective surgeries should still be delayed on a personalized basis to maximize postoperative outcomes. We outline a framework for stratifying the individual COVID-19 patient’s fitness for surgery based on the symptoms and severity of acute or convalescent COVID-19 illness, coagulopathy assessment, and acuity of the surgical procedure. Although the most common manifestation of severe acute respiratory syndrome coronavirus 2 (SARS-CoV-2) infection is COVID-19 pneumonitis, every system in the body is potentially afflicted by an endotheliitis. This endothelial derangement most often manifests as a hypercoagulable state on admission with associated occult and symptomatic venous and arterial thromboembolisms. The delicate balance between hyper and hypocoagulable states is defined by the local immune-thrombotic crosstalk that results commonly in a hemostatic derangement known as fibrinolytic shutdown. In tandem, the hemostatic derangements that occur during acute COVID-19 infection affect not only the timing of surgical procedures, but also the incidence of postoperative hemostatic complications related to COVID-19-associated coagulopathy (CAC). Traditional methods of thromboprophylaxis and treatment of thromboses after surgery require a tailored approach guided by an understanding of the pathophysiologic underpinnings of the COVID-19 patient. Likewise, a prolonged period of risk for developing hemostatic complications following hospitalization due to COVID-19 has resulted in guidelines from differing societies that recommend varying periods of delay following SARS-CoV-2 infection. In conclusion, we propose the perioperative, personalized assessment of COVID-19 patients’ CAC using viscoelastic hemostatic assays and fluorescent microclot analysis.

## Introduction: Perioperative COVID-19 and Postoperative Morbidity and Mortality

The global impact of the coronavirus disease 2019 (COVID-19) pandemic on surgical procedures was unprecedented (1, 2). After the induction of the national state of emergency in the United States in March 2020, the Centers for Medicare and Medicaid Services (CMS), the American College of Surgeons (ACS), and other international surgical societies recommended postponing, minimizing, and cancelling elective surgical procedures based largely on SARS-CoV-2 transmissibility precautions (3–5). During this time, there was a 50% reduction in the number of surgical procedures performed relative to baseline (1). Five weeks later, a joint recommendation was published that advised the resumption of elective surgical procedures (6). As the demand for elective procedures increased, clinicians were faced with a growing number of patients still recovering from COVID-19, and surgeons had little available evidence for the optimal timing of surgery and postoperative prognosis for those recovering from acute and/or convalescent COVID-19 illness (7).

As the pandemic continued and data accrued, postoperative morbidity and mortality were confirmed to be significantly higher for patients with perioperative COVID-19 infection (8). Perioperative COVID-19 infection correlated with higher rates of postoperative pulmonary complications and venous thromboemboli (VTE) (e.g., pulmonary emboli [PE] and deep venous thromboses [DVT]) (9). Notably, those with ongoing symptoms at the time of operation—regardless of length from diagnosis/onset of COVID-19 symptoms—have a significantly greater risk of VTE and associated mortality. VTE has been independently associated with 30-day postoperative mortality (odds ratio 5.4 [95%CI 4.3–6.7]) (9).

One prospective cohort study by the COVIDSurg Collaborative evaluated >140,000 surgical patients from 116 countries who underwent elective or emergency surgery during October 2020 (10). Surgical patients with preoperative SARS-CoV-2 infection were compared to those without previous SARS-CoV-2 infection. There was a significant increase in postoperative pulmonary complications and mortality in patients with a recent diagnosis of SARS-CoV-2 infection (10). The calculated 30-day mortality rates were stratified by time between diagnosis of SARS-CoV-2 infection and surgery (groups included those diagnosed 0–2, 3–4, 5–6, and ≥7 weeks prior to surgery). For those patients without SARS-CoV-2 infection, the adjusted 30-day mortality rate was 1.5%. For those patients diagnosed with SARS-CoV-2 infection, 30-day postoperative mortality rates were 9.1% for 0–2 weeks; 6.9% for 3–4 weeks; 5.5% for 5–6 weeks; and 2.0% at ≥7 weeks between diagnosis and surgery. Surgery performed ≥7 weeks after SARS-CoV-2 diagnosis was associated with a similar mortality risk to baseline for controls (odds ratio 1.5 [95%CI 0.9–2.1]). Further stratifying this analysis to those patients with ongoing symptoms at time of surgery, it was noted that even after a ≥7 week delay from SARS-CoV-2 infection diagnosis, these symptomatic convalescent patients had a higher mortality than patients whose symptoms had resolved or who had been asymptomatic at operation (6.0% for ongoing symptoms, vs. 2.4% for resolved symptoms, vs. 1.3% for controls).

Consequently, the authors recommended that surgery be delayed for at least seven weeks following SARS-CoV-2 infection, and concluded that patients with ongoing symptoms at seven weeks may benefit from even longer delay (10). A recent multidisciplinary consensus statement aligned with these study conclusions, stating that elective surgery should be scheduled seven or more weeks after the diagnosis of COVID-19 for patients with asymptomatic or transient SARS-CoV-2 infection, while patients with persistent symptoms (i.e., “Long COVID”) remain at elevated risk for postoperative morbidity and mortality even after seven weeks. It is therefore recommended to stratify patient risk in delaying surgery beyond seven weeks on a personalized basis (11). In one systematic review including >250,000 patients, more than half had post-acute sequelae of COVID-19 at six months follow-up (12). Clearly, there is a significant need to risk stratify this patient population on an individualized basis.

The most recent American Society of Anesthesiologists (ASA) and Anesthesia Patient Safety Foundation (APSF) joint recommendation for the delay of surgery have been divided into four blocks of time based upon the symptoms and severity of acute COVID-19 illness:

- “Four weeks for an asymptomatic patient or recovery from only mild, non-respiratory symptoms
- Six weeks for a symptomatic patient (e.g., cough, dyspnea) who did not require hospitalization
- Eight to 10 weeks for a symptomatic patient who is diabetic, immunocompromised, or hospitalized
- Twelve weeks for a patient who was admitted to an intensive care unit due to COVID-19 infection”

The ASA and APSF joint recommendation further states: “These timelines should not be considered definitive; each patient’s preoperative risk assessment should be individualized, factoring in surgical intensity, patient co-morbidities, and the benefit/risk ratio of further delaying surgery” (13). Specifically, these recommendations have not considered the differing degrees of coagulopathy associated with COVID-19 variants. Additionally, there is little guidance on elective surgery for patients afflicted with Long COVID, and there are few proposals for preoperative laboratory measurements (e.g., D-dimer, fibrinogen, ferritin, lactate dehydrogenase (LDH), and other clinical and laboratory markers of severity of disease) to guide surgeons and anesthesiologists for the appropriate timing of surgery (14, 15).

Likewise, sparse guidance exists for the postoperative thromboprophylaxis of this patient group. In a large study of 11,249 patients from the first COVID-19 surge in the Spring of 2020 in New York City, 1.7% of all patients discharged from the hospital had VTE or arterial thromboembolism (16). This study also reported that prophylactic anticoagulation with either enoxaparin or rivaroxaban engenders a 46% reduction in clotting disorders (16). However, the unique pathophysiological aspect of COVID-19 pneumonitis is that these patients may bleed and clot at the same time when anticoagulated (17). It was reported that 1.73% of discharged patients suffered major bleeding within 90 days of discharge while only 13.2% of the entire cohort was administered thromboprophylaxis (16).

**Figure 1:**
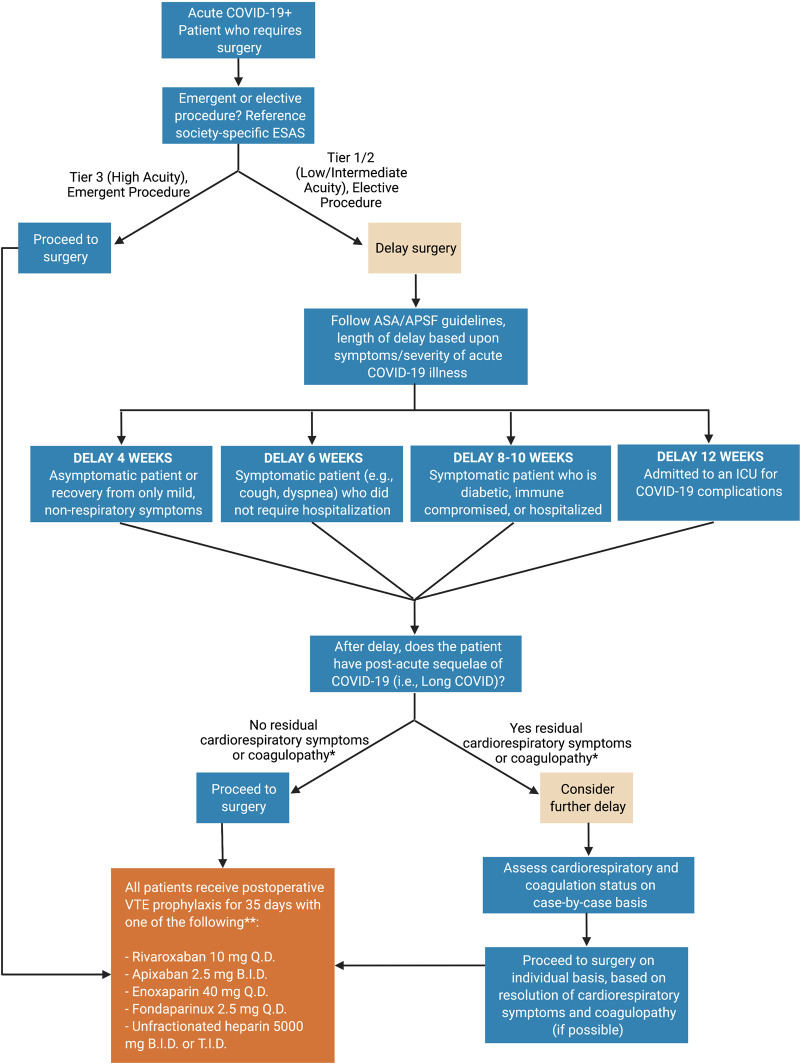
**COVID-19 patient surgical fitness flow chart.** The surgeon assesses the acuity of the procedure using the specialty-specific Elective Surgery Acuity Scale (ESAS). If the procedure is of low/intermediate acuity and can be delayed, the length of delay may follow American Society of Anesthesiologists’ recommendations. After the delay, timing of surgery is a function of both the necessity of surgery and the degree of recovery of respiratory and hemostatic derangement post-COVID-19. Post-operatively, due to a high degree of hypercoagulability in many convalescent COVID-19 patients, standard pharmacologic VTE prophylaxis may be considered in all these patients. Created using BioRender.com. *Cardiorespiratory symptoms such as shortness of breath, chest pain, and fatigue are common in patients with convalescent COVID-19 (12, 13, 18, 19). Coagulopathy, including both hyper and hypocoagulable states, should be assessed pre- and post-operatively using fibrinogen levels, platelet count, and viscoelastic hemostatic assays (e.g., thromboelastography (TEG) and rotational thromboelastometry (ROTEM)). The definition of hyper and hypocoagulability are a function of institutional preference. **This is not an exhaustive list of anticoagulant agents, nor a comprehensive dosing regimen. For inpatients acutely ill with COVID-19, those with VTE, those who undergo emergent surgery, or those at risk for hemorrhage, personalized titration of anticoagulation with adjunctive viscoelastic hemostatic assays is recommended (17, 20–23). VTE prophylaxis for postoperative patients with acute or convalescent COVID-19 is not specifically addressed in recent expert consensus statements or society recommendations (24–26). As such, this recommendation is the opinion of the authors and should be judiciously applied taking into account each individual patient’s risk and benefit to anticoagulant therapy.

Together these findings offer surgeons the opportunity to familiarize themselves with the unique hemostatic derangement of CAC affecting postoperative outcomes worldwide. A basic flow chart for triaging acute and convalescent COVID-19 patients based upon recent ASA and surgical guidelines for the timing of surgery and postoperative VTE prophylaxis is presented in **Figure 1**. Clear data demonstrates unvaccinated individuals with COVID infection procure a higher risk of thrombosis and more severe illness. Breakthrough infections in vaccinated individuals have less thrombotic risk and lower risk of severe illness (27). Following the ASA guidelines, patients’ length of delay for surgery should be based on the severity of illness regardless of vaccination status.

The impact of COVID-19 on immune system-related complications in surgical patients is a crosstalk between immunology and coagulation at the level of the endothelium. This is more than a theoretical concept; rather, it is an important pathophysiologic understanding of the unique challenges of operating on patients who have either recent or remote infections with the SARS-CoV-2 virus. Few guidelines exist concerning the timing and nature of this dual threat of bleeding and clotting that occur in a surgical patient with COVID-19 (28, 29). This review will address the pathophysiologic underpinnings of COVID-19 as it relates to surgical timing and thromboprophylaxis, followed by a specific application of these immune-thrombotic changes to perioperative and postoperative management of patients.

## COVID-19-Associated Coagulopathy (CAC)

Recent consensus and guidelines exist, but they lack granularity to assist the surgeon for optimizing the timing of elective surgery and guiding postoperative thromboprophylaxis for their individual convalescent COVID-19 patients. The long-term effects of COVID-19 infection are still under investigation. Hence, there are limited studies or specific guidelines aiding the surgeon in weighing risks and benefits of elective surgery for patients post-recovery from COVID-19. Persistent complications of COVID-19 infection after recovery are a product of the vast endothelial damage induced by SARS-CoV-2 (29). It is pertinent for the surgeon and anesthesiologist to understand the mechanisms of this endotheliitis to individualize care for their convalescent COVID-19 patients in this time of limited evidence-based guidance.

### Pathophysiology of CAC: A Labile Balance Between Fibrinolysis and Fibrinolytic Shutdown

**Figure 2:**
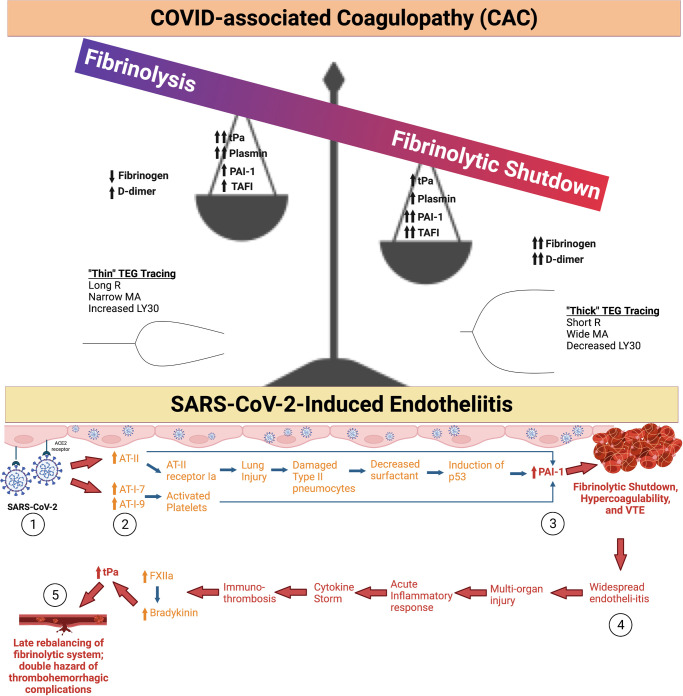
**COVID-19-associated coagulopathy (CAC) and Fibrinolytic Shutdown. (1)** The SARS-CoV-2 Spike protein binds with angiotensin converting enzyme 2 (ACE2), which causes internalization of the receptor and virus into the host cell. **(2)** In turn, ACE2 is not available for breakdown of angiotensin II (AT-II) leading to its build-up. AT-II causes subsequent lung injury and increases in plasminogen activated inhibitor 1 (PAI-1). Angiotensin I-7 (AT-I-7) and angiotensin I-9 (AT-I-9) are increased by other pathways involving activated platelets, but also lead to increased levels of PAI-1. **(3)** Increased circulating PAI-1 leads to fibrinolytic shutdown, a hypercoagulable state characterized by increased fibrin deposition and thrombus formation. **(4)** Among other mechanisms, widespread endotheliitis and microthrombosis can cause multi-organ injury in patients with severe COVID-19 illness. This exacerbates the acute inflammatory response, often causing a cytokine storm and worsening immuno-thrombosis. Inflammatory cytokines activate Factor XII (FXII), which subsequently increases bradykinin levels. Bradykinin increases tissue plasminogen activator (tPa) levels. **(5)** This subacute rebalancing of the fibrinolytic system predisposes the COVID-19 patient to both thrombosis and hemorrhage as a function of local endothelial derangement. This is particularly the case for hospitalized COVID-19 patients who are anticoagulated; these patients’ anticoagulant regimen must be judiciously titrated on a personalized basis (17, 29, 31). The spectrum of fibrinolysis is depicted as a balance whereby the predominate hypercoagulable fibrinolytic shutdown phenotype is portrayed as a balance with high levels of PAI-1, thrombin activatable fibrinolysis inhibitor (TAFI), fibrinogen, D-dimer, and lower levels of tPa and plasmin. The opposite occurs at the opposite end of the fibrinolysis spectrum when fibrinolysis predominates, which occurs much less frequently in CAC. Created using BioRender.com

Immuno-thrombosis is often described as the dysregulated interaction between innate immunity and coagulation (30–32). A reported 30-80% of COVID-19 ICU patients endure thrombotic complications during their clinical course (33–36). The most common thrombotic complication in COVID-19 patients is VTE, which occurs in 15-85% of patients (37). Although the exact mechanism behind CAC is poorly understood, endotheliitis likely incites the hypercoagulable state (**Figure 2**) (38–42). The SARS-CoV-2 virus directly invades endothelial cells through the angiotensin converting enzyme 2 (ACE2) receptor. This leads to localization of viral inclusion bodies in the lungs, liver, small intestines, and kidneys, shedding of endothelial glycocalyx proteins, and decreases in the level of heparanase-2. This results in a progression from normal hemostasis to a relatively antifibrinolytic state (31, 38, 43–45). The SARS-CoV-2 viral saturation of ACE2 increases circulating levels of angiotensin II, leading to increased production of the important antifibrinolytic mediator, plasminogen activator inhibitor 1 (PAI-1) (46–48). Elevated PAI-1 and thrombin-activatable fibrinolysis inhibitor (TAFI) levels in the alveolar space have been identified in COVID-19 patients (46, 49–51). Elevated tissue plasminogen activator (tPa) levels have also been noted in COVID-19 patients. This tPa release may be due to malperfusion secondary to microvascular fibrin deposition. Despite this increase in tPa, pro-thrombosis persists in these patients, suggesting that elevated PAI-1 and TAFI overwhelm the action of tPa, resulting in microvascular deposition of fibrin (52). These factors contribute to the disruption of the tightly regulated balance between thrombosis and fibrinolysis in COVID-19 patients, leading to what is known as “fibrinolytic shutdown” (17, 46, 50, 52–58). When combined with elevated thrombin generation, it leads to adverse complications such as a type of adult respiratory distress syndrome (ARDS), multisystem organ failure, and mortality (29, 59–62). This unique type of ARDS is often associated with a localized hypercoagulability of the pulmonary vasculature due to abnormal deposition of fibrin and development of microthrombosis (63–66). Postmortem analyses confirm a similar presentation in patients with COVID-19-induced ARDS (67–70). This localized hypercoagulability is partially induced by inflammation of tissue, leading to elevated tissue factor (TF) production by epithelial cells and alveolar macrophages which results in the deposition of fibrin and the development of microthrombosis (46, 71). Thromboses are additionally potentiated by a deficient fibrinolytic response primarily induced by the overexpression of PAI-1 from activated platelets and endothelial cells (31, 72, 73).

Primary hemostasis, which is accomplished via activated platelets at the site of endothelial injury, contributes to the hypercoagulable state of the COVID-19 patient. Overwhelming platelet activation is driven by many stimuli, and significantly facilitated by increased levels of von Willebrand Factor (VWF), as well as activation of the enzymatic (intrinsic) clotting pathway. COVID-19 patients have increased activation of circulating platelets as demonstrated by elevated soluble P-selectin as well as markers for endothelial activation (74). A direct stimulatory role of SARS-CoV-2 Spike protein on the COVID-19 patient’s platelets has also been described (75). Severe COVID-19 illness correlates not just with platelet activation but is also associated with enhanced platelet-monocyte aggregation. Finally, ICU admitted COVID-19 patients’ platelets induce P-selectin and αIIb/β3-dependent monocyte TF expression which may augment inflammation and hypercoagulability (76).

**Figure 3:**
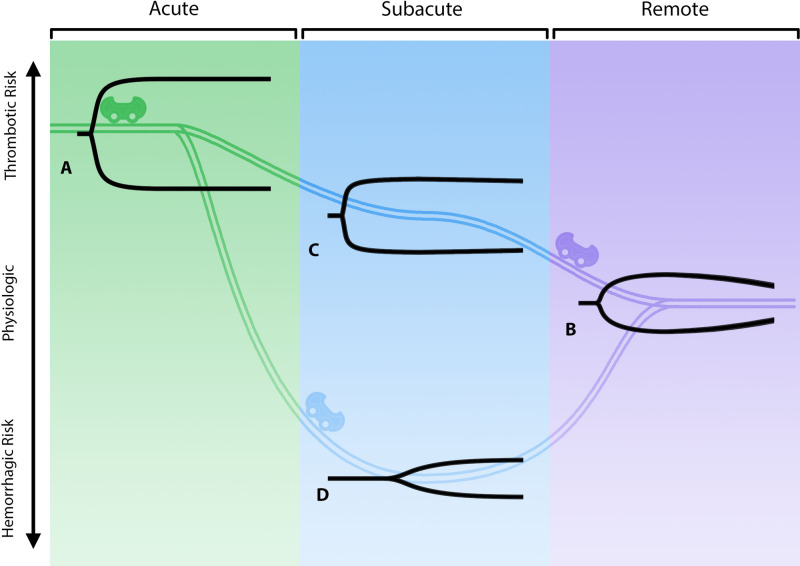
**The “rollercoaster” phenomenon of COVID-19-associated coagulopathy.** The three stages of COVID-19-associated coagulopathy and their relationship to the immuno-thrombotic derangement caused by the cytokine storm are represented above by viscoelastic hemostatic assays (e.g., thromboelastography [TEG]). (**A**) represents the acute fibrinolytic shutdown phenotype (short R, steep α, thick MA, no lysis). (**B**) represents the eventual evolution to a normal physiologic fibrinolytic phenotype (normal R, α, MA, and LY30). (**C**) represents the TEG in evolution without anticoagulation and the subacute recovery stage where the patient is less hypercoagulable (parameters intermediate between acute and remote). (**D**) represents the patient with or without anticoagulant with a hypocoagulopathic phenotype due to hypofibrinogenemia and/or a thrombocytopenia in spite of persistent fibrinolytic shutdown (long R, flat α, narrow MA, no lysis) (17, 20, 77, 79–81). Created using Adobe Illustrator.

Together, these aberrancies in hemostasis mediated by platelets and reduced fibrinolysis (documented by whole blood viscoelastic hemostatic assays [VHAs], thromboelastography [TEG] and rotational thromboelastometry [ROTEM]) inevitably lead to uncontrollable hypercoagulopathy (76–78)*.* The TEG/ROTEM evaluates the patient’s position along the hemostatic spectrum from hypo to hypercoagulable states. The tracings resemble a shovel, whereby the length of the handle reflects coagulation factor competence, the slope of the blade reflects fibrinogen concentration, the thickness of the blade reflects clot contraction mediated by fibrin-platelet interaction, and the tapering end of the blade represents fibrinolysis. Therefore, a characteristic early COVID-19 patient with a hypercoagulable state presents with a short handle, a steep slope, and a thick blade with no tapering of the blade, which represents enhanced effect of coagulation factors, increased fibrinogen concentration, increased fibrin/platelet function, and fibrinolytic shutdown. At the other end of the spectrum lies the hypocoagulable state as manifested by a long handle, flat slope, thin blade, with significant tapering. In **Figure 3**, examples of this paradigm are represented along various stages of this spectrum of coagulopathies associated with COVID-19.

The hypercoagulability of COVID-19 patients is clearly complex and multifactorial, but understanding these complex mechanisms allows for the pursuit of specific treatment strategies to improve patient outcomes. Management is complicated because of fibrinolytic shutdown due to the reduction of natural fibrinolysis at the level of the endothelium. While a multitude of studies have shown the detrimental effect of fibrinolytic shutdown on COVID-19 patient outcomes, bleeding complications such as gastrointestinal bleeding and hemorrhagic stroke are also common, especially in patients on therapeutic anticoagulation (17, 79, 80, 82–86). These patients tend to clot and bleed at the same time, a function of the “rollercoaster” phenomenon (77).

### Timing of Elective Surgery for Acute and Convalescent COVID-19 Patients

Even for those patients without comorbidities or illness requiring hospitalization, a large number of convalescent COVID-19 patients at four months post-infection are reported to have one or more residual organ system impairments as defined by quantitative magnetic resonance imaging (MRI) T1 mapping (12, 87). This phenomenon is termed Long COVID (or post-acute sequelae of COVID-19 [PASC]) and can involve any organ system (88). Hypercoagulability and VTE have been described as potential complications in Long COVID patients (88). In the convalescent COVID-19 patient, it is important to understand that the timing of surgery is a function of where the patient lies within the context of their individual immuno-thrombotic state (77).

Further research is necessary for determining potential biomarkers which may allow surgeons to weigh the risks and benefits of elective surgery. Multiple studies have shown persistently elevated D-dimer, fibrinogen, C-reactive protein (CRP), interleukin (IL)-4, IL-6, tumor necrosis factor (TNF)-α, interferon (IFN)-γ-induced protein 10, factor VIII, VWF, and plasma soluble thrombomodulin in patients up to six months post-recovery from COVID-19 (81, 89–94), indicating that these may serve as potential biomarkers for assessing the risk of postoperative complications. Hypercoagulability markers are often associated with postoperative complications in non-COVID-19 perioperative settings (e.g., PAI-1, TAFI, increased fibrinogen and platelet counts) (7, 95–97). Research on these and other biomarkers in assessing postoperative morbidity and mortality in convalescent COVID-19 patients undergoing elective surgery, however, is lacking. Future studies are required in order to determine the most prognostic biomarkers for evaluating this patient population (88).

### Treatment and Thromboprophylaxis for the Postoperative COVID-19 Patient

COVID-19 and the subsequent damage to endothelial cells result in a progression from hemostasis to a relatively prothrombotic/ antifibrinolytic state (31, 38, 43–45). Clinical data demonstrate significant thrombophilia with fibrinolysis shutdown being a primary component, regulated by higher circulating levels of antifibrinolytic factors, namely PAI-1 and TAFI. Yet, coagulation is a rigorously regulated process: fibrinolytic and thrombotic pathways are continuously competing against each other such that neither hemorrhage nor thrombosis is favored under normal physiological conditions (98). Therapeutic strategies for COVID-19 patients that utilize these findings, including the use of fibrinolytics, have been under examination and show potential for improving patient outcomes. For patients being treated with COVID-19, therapeutic anticoagulation is the standard regimen for patients with macrothrombosis. Furthermore, the use of anticoagulant heparinoids is advised for all COVID-19 patients (99). The use of tPa has been suggested for patients with severe ARDS caused by COVID-19 (100). In these studies, the use has been late in the disease course. Theoretically, if tPa is used earlier during the hypercoagulable stages of the disease, the fibrinolytic and therapeutic effect may be greater than those reported in the recent literature. Also, when administered later in the course of the disease—as the cytokine storm recedes—the patients may be prone to hemorrhage similar to anticoagulated COVID-19 patients (17, 101).

Therapeutic anticoagulation and thromboprophylaxis for the hospitalized COVID-19 surgical patient vary substantially. Recommendations have been made from adherence to standard VTE prophylactic guidance to intermediate or therapeutic dosing for moderately ill COVID-19 patients (85, 86). The preferred agents have been enoxaparin with anti-Xa monitoring or unfractionated heparin with either activated partial thromboplastin time (aPTT) or anti-Xa guidance. This heterogeneity is based on large multiplatform randomized controlled trials (RCTs) as well as society consensus statements. Recent guidelines and consensus statements have considered conditional support for therapeutic and/or intermediate heparinoid thromboprophylaxis for moderately ill COVID-19 patients not requiring high-flow oxygen (**Table 1**) (24–26, 85, 86, 99, 102–104). The importance of this foundational literature has been highlighted by observations that clinicians find it difficult to practice medicine during the COVID-19 pandemic bereft of RCTs to guide treatment (105). Therefore, reliance on categorization of hemostatic phenotype by VHAs fulfills the criteria of personalized-based medicine as a guidance for anticoagulation and thromboprophylaxis for the COVID-19 surgical patient.

**Table 1:** Salient society recommendations on anticoagulant dosing for acute COVID-19 patients.

Society	Outpatient	Inpatient	Intensive Care Unit	Post-discharge
American Society of Hematology (February 2, 2022) (24)	Thromboprophylaxis not recommended	For patients without VTE, standard prophylactic dose with LMWH or UFH	For patients without VTE, standard prophylactic dose with LMWH or UFH	Thromboprophylaxis not recommended
American College of Chest Physicians (CHEST) Guideline (February 12, 2022) (25)	Not addressed	Conditional recommendation: For patients without VTE, therapeutic dose with LMWH or UFH	For patients without VTE, standard prophylactic dose with LMWH or UFH	Not addressed
National Institutes of Health (NIH) Guidelines (February 24, 2022) (26)	Not addressed	Therapeutic dose heparin only for patients hospitalized on low-flow oxygen For hospitalized patients without VTE and not requiring oxygen or requiring high-flow device or NIV, standard prophylactic dose with LMWH or UFH	For patients without VTE, standard prophylactic dose with LMWH or UFH	Thromboprophylaxis not recommended

*Abbreviations: LMWH, low molecular weight heparin; UFH, unfractionated heparin; VTE, venous thromboembolism.*

For elective cases, a high incidence of patients discharged from the hospital have VTEs within 90 days of discharge from COVID-19 (16). In addition, a subsection of the population who have COVID-19 and have undergone surgery have a markedly elevated rate of VTEs (9). Because of the absence of specific RCTs recommending postoperative DVT prophylaxis in this group of patients, use of Elective Surgery Acuity Scale (ESAS) guidelines as well as co-morbidities and adjunctive VHA analysis would allow for postoperative DVT prophylaxis similar to that which has been recommended for the post-COVID discharged patient. Specifically, the recommendations vary from 10 mg rivaroxaban for 35 days, 2.5 mg apixaban b.i.d. for 35 days, or for patients with normal renal function, 40 mg enoxaparin for 35 days (102, 106, 107). Specific co-morbid analysis using conventional coagulation tests with adjunctive TEG-guided anticoagulation for COVID-19 patients with and without macrothrombosis has demonstrated the ability of the TEG to assist in the prediction of bleeding and clotting in this group of patients (17, 108). Similarly, the large RCTs which have demonstrated therapeutic benefit of heparinoids in moderately ill COVID-19 patients would be refined by the addition of VHAs to guide anticoagulant therapy (17, 85, 86, 109).

As has been shown, so-called “intermediate” and therapeutic anticoagulation has been found to protect patients with moderately severe COVID-19 pneumonitis (86). Surgeons will find themselves during the acute and subacute period considering emergent and semi-emergent procedures for patients on anticoagulation not just for known macrothromboses but also for the specific antiviral and antiinflammatory functions of heparinoids in these patients (85, 86, 109). In addition to conventional coagulation tests, anticoagulation for these patients is facilitated by adjunctive hemostatic monitoring with VHAs (17, 20–22).

The sequelae of Long COVID may be a function of residual endothelial damage from the SARS-CoV-2 endothelial cell invasion (88, 110). As such, thromboprophylaxis for postoperative convalescent COVID-19 patients should draw from strategies to manage acute COVID-19 patients along with the strategies for non-COVID-19 patients undergoing elective surgery.

### Viscoelastic Hemostatic Assays and Plasma Fluorescent Microclot Analysis as Precision-based Tools to Contextualize COVID-19-associated Immuno-thrombosis

Surgeons—because of their more than half century experience with TEG/ROTEM to diagnose and treat coagulopathies in liver transplantation, cardiac surgery, and trauma—have been at the forefront of pathophysiologic investigations of CAC (111). Because of surgeons’ historical experience on the intricacies of hemostasis, surgeons have employed TEG/ROTEM to guide and personalize anticoagulation during the COVID-19 pandemic (20–22, 28, 29, 101, 112). TEG/ROTEM have also been used to profile postoperative patients at risk for VTE (23, 113–116). This background in using TEG/ROTEM to predict a patient’s fibrinolytic phenotype prepares the surgeon to manage CAC patients. Given the significant variation in risk of the development of either a hypocoagulopathic- or hypercoagulopathic-related complication in the perioperative period as a function of the severity of COVID-19 illness, it is difficult to rely on arbitrary standards regarding the timing of surgery and the nature of anticoagulation and thromboprophylaxis in this group of patients (17, 28, 29, 31, 108).

**Figure 4:**
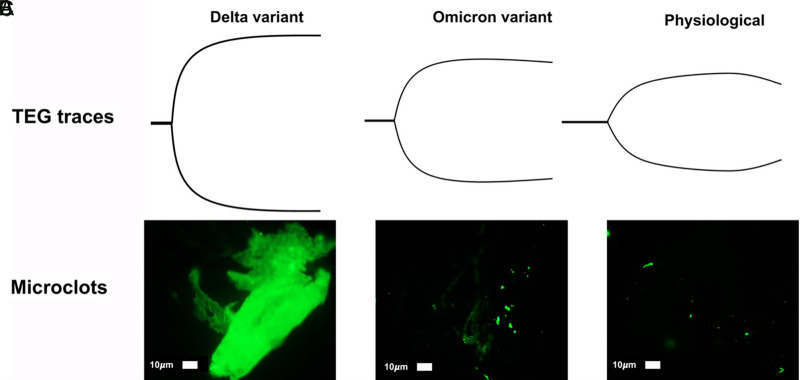
**Prototype spectrum of COVID-19-associated coagulopathy used to assess the patient’s coagulopathy with thromboelastography (TEG) and plasma microclot analysis.** Shown above is an example of correlation between the most hypercoagulable delta (B.1.617.1) variant, intermediate hypercoagulable omicron (B.1.1.529) variant, and physiologic TEGs with corresponding plasma microclot analyses. Using Thioflavin-T as a fluorescent marker specifically for microclot staining, fluorescence microscopy demonstrates microclots in plasma with representative examples of different degrees of microclot formation as related to delta (**A**), omicron (**B**), and physiologic (**C**) TEGs. Previously described, Stage 1 to 4 is a qualitative numerical scoring system where a score of 4 is given for significant and widespread microclot formation and a score of 1 is given for minimal microclot formation (78, 119). In this figure, delta variant (**A**) equals stage 4, omicron (non-hospitalized patient) (**B**) is stage 3, normal physiologic non-infected individual (**C**) is stage 2. A hypocoagulable state either from anticoagulation of the COVID-19 patient or due to naturally acquired disease from COVID-19-associated coagulopathy is stage 1 (not pictured). Patients with Long COVID also demonstrate microclot formation, which may be used to assess platelet dysfunction and surgical candidacy in this group of patients (77, 78, 119). The omicron variant (BA.1 sub-lineage) was detected by polymerase chain reaction (PCR) based on S-gene target failure (SGTF) as a proxy for variant status (https://www.science.org/doi/10.1126/science.abn4543) (120). The TaqPath COVID-19 Combo Kit^®^ (Thermo Fisher Scientific) identifies SARS-CoV-2 infections by detecting 3 viral gene regions, *E*, *RdRP*, and *N* gene. The laboratory reported positive cycle threshold of 32 cycles for both the *E* gene, 35 for RdRP and 34 for the *N* gene. Positive *N* gene with negative *S* gene (SGTF) is an acceptable proxy of the BA.1 variant when the gold-standard whole genome sequencing is not done. The PCR result combined with reported case counts and known genomic epidemiology of the 4^th^ wave in South Africa has been used to track changes in transmission over time for the BA.1 variant (121, 122).

The recent evolution of the less virulent but more infectious variants, such as the omicron (B.1.1.529), may render guidelines regarding the timing and nature of therapeutic anticoagulation and thromboprophylaxis for the surgical patient less applicable. Although there is no data directly demonstrating lower risk of thrombosis in patients infected with omicron versus other more virulent variants, thrombosis risk in COVID-19 patients is in large part a function of illness severity (117, 118). By logical extension of this evidence, it is likely omicron infection carries less thrombotic risk compared to more virulent variants such as alpha or delta. Age, co-morbidities, and overall clinical picture are paramount to consider when deciding to what degree the individual patient should be anticoagulated. There are no plasma-based laboratory tests which allow for prediction of the likelihood of perioperative complications regarding coagulation for the surgical patient with either acute or remote COVID-19 infection. Historically, VHAs have been used to predict both bleeding and clotting in patients undergoing liver transplantation, cardiac surgery, trauma surgery, and most recently, bleeding and clotting obstetric patients. Most recently, adjunctive TEG has demonstrated prediction of VTE in critically ill COVID-19 patients (20–23). However, categorizing patients’ fibrinolytic and coagulopathic phenotype with TEG/ROTEM, as has been done by surgeons for decades, may allow the surgeon to better predict the development of perioperative clot and to manage pre- and post-surgical clots more effectively in the COVID-19 patient. In addition, platelet activation can be graded with plasma microclot density analysis, which has demonstrated that Long COVID patients have a persistent coagulopathy and increased microclot formation (119). Together, TEG/ROTEM with microclot analysis may predict the surgical patient’s position in the hemostatic and fibrinolysis spectrum in the absence of evidenced biomarkers. **Figure 3** illustrates the ‘rollercoaster’ phenomenon of hospitalized COVID-19 patients as characterized by TEG/ROTEM in the acute and subacute periods, as well as the remote period after discharge (77). **Figure 4** provides a prototype spectrum of CAC wherein the patient’s individual coagulopathy may be modified by the COVID-19 variant and is characterized by TEG/ROTEM and plasma microclot analysis. Studies are in progress regarding microclot analysis as an enhanced method of hemostatic grading (78).

## Surgical Societies’ Recommendations for the COVID-19 Patient’s Timing of Surgery and Thromboprophylaxis

Emergency surgery in the acute period of COVID-19 infection requiring hospitalization is associated with high mortality (8, 123). These increases in mortality have been observed across various surgical specialties (123). As a result, there are surgical specialty-specific recommendations regarding the timing of surgery for patients with symptomatic and asymptomatic SARS-CoV-2 infection. These recommendations are varied and a function of institutional preference and expert consensus. **Table 2** and the text below summarizes the literature and recommendations of the leading surgical and obstetric organizations regarding the timing and administration of thromboprophylaxis for patients with acute and remote SARS-CoV-2 infection.

**Table 2:** Society recommendations on timing of surgery and thromboprophylaxis in acute and convalescent COVID-19 patients. Note this table is not exhaustive, but reflects the diverse and heterogeneous recommendations regarding the timing of surgery and anticoagulation for this patient population.

Discipline	Association	Recommendations
Anesthesia	American Society of Anesthesiologists (13)	Timing of elective surgery should be delayed based on the symptoms and severity of acute COVID-19 illness. From time of diagnosis: Four weeks for an asymptomatic patient or those recovering from mild, non-respiratory symptoms Six weeks for a patient with respiratory symptoms who was not hospitalized Eight to 10 weeks for a symptomatic patient who is immunocompromised, diabetic, or was hospitalized Twelve weeks for a patient who was admitted to an ICU for COVID-19 complications
Obstetrics	American College of Obstetricians and Gynecologists (124)	Surgery may be delayed when a patient’s health would not be harmed. Thrombosis risk may be increased with COVID-19 infection Pregnant patients hospitalized for severe COVID-19 receive prophylactic dose anticoagulation unless contraindicated
	American Society for Reproductive Medicine (125)	Procedures should not be delayed if patient health is at risk; triage into four periods of surgery: Cannot be delayed (e.g., hysteroscopy) Delay up to four weeks (e.g., colposcopy, LEEP) Delay four to twelve weeks (e.g., TVUS, vulvar biopsy) Delay beyond twelve weeks (e.g., Botox)
	Royal College of Obstetricians and Gynecologists (126)	Thromboprophylaxis should still be offered and administered as normally indicated during the COVID-19 pandemic, should be continued if patient contracts COVID-19 Pregnant women with COVID-19 should be given prophylactic LMWH, unless birth is expected within 12 hours or there is significant risk of hemorrhage Pregnant women hospitalized with COVID-19 should be offered thromboprophylaxis for 10 days following discharge and longer with thrombophilic co-morbidities Postpartum patient hospitalized for COVID-19 infection should be offered thromboprophylaxis during hospitalization and for at least 10 days after discharge, and up to 6 weeks of thromboprophylaxis for thrombophilic co-morbidities Avoid general anesthesia for at least 7 weeks after COVID-19 infection
Orthopaedics	American Academy of Orthopedic Surgeons (127)	Reschedule elective surgery for individuals with ongoing COVID-19 Delay elective surgery based on local capacity to care for COVID-19 patients
	American Association for Hand Surgery (128)	Delay surgery based on local capacity to care for COVID-19 patients
	American Association of Hip and Knee Surgeons (129)	Prioritize surgery based on clinical need
	American Orthopaedic Association (130)	Delay surgery based on clinical need and institutional resource availability
	European Hip Society and European Knee Associates (131)	For infected otherwise healthy individuals, delay elective arthroplasty by 6 weeks For infected patients with one or more co-morbidity, delay surgery by 8 weeks
General Surgery	American Society for Metabolic and Bariatric Surgery (132)	Delay surgery based on risk factors, local viral prevalence, and institutional resource availability on an individual basis
	American Society of General Surgeons (133), American College of Surgeons (134, 135)	Prioritize surgery based on risk factors and patient benefits on an individual basis Discernment process is streamlined at an institutional level, with final input coming from the surgeon Defer to Centers for Medicare & Medicaid Services (CMS) tiered framework for urgency of procedures based upon Elective Surgery Acuity Scale (ESAS)
	Centers for Medicare & Medicaid Services (CMS) (136)	Three-tiered system to decide whether to postpone surgery based upon two factors: acuity of procedure and the health of the patient Tier 1, low acuity surgery – postpone (e.g., carpal tunnel release, routine colonoscopy) Tier 2, intermediate acuity surgery – consider postponing (e.g., arthroplasty, elective angioplasty + stent) Tier 3, high acuity surgery – do not postpone (e.g., trauma, cancers, neurosurgery)
	American Society of Transplant Surgeons (137)	A positive PCR test should result in delay of procedure A period of 2-4 weeks of negative serology is required for waitlist reactivation of immunosuppressed candidates after COVID-19 contraction
	Society of American Gastrointestinal Endoscopic Surgeons (138)	Delay surgery based on risk factors (age and potential comorbidity) and institutional resource availability Enhanced CMS guidelines: for example, for T3 or higher gastric cancers neoadjuvant chemotherapy can be an alternative, allowing surgical delay up to 3-4 months but is dependent upon the rate of disease progression
Neurosurgery	European Association of Neurosurgical Societies (139)	Elective Surgery Acuity Scale (ESAS) adapted to neurosurgical procedures There are three tiers of procedures: Tier 1, low acuity surgery: postpone (e.g., benign intracranial tumors) Tier 2, intermediate acuity surgery: postpone if possible (e.g., AV malformation, unruptured aneurysm) Tier 3, high acuity surgery: do not postpone (e.g., malignant brain or spine tumor)
Cardio-toracic & Vascular Surgery	Canadian Society of Cardiac Surgeons (140)	Details three phases of ‘ramping up’ cardiac surgery case volume based on hospital capacity Emphasizes which surgical procedures are emergent or can be delayed for medical or percutaneous interventions
	Society of Thoracic Surgeons (141)	Describes four tiers of operative capacity, wherein each tier details essential procedures and which should be deferred
	Society for Vascular Surgery (142)	Links to worldwide society guidelines are provided including adaptations of CMS and ACS protocols
	European Society for Vascular Surgery Management Guidelines for Acute Limb Ischemia (ALI) (143)	Open and endovascular interventions for acute limb ischemia (ALI) for patients with COVID-19 have a mortality rate of 20.4% Therapeutic anticoagulation with intravenous unfractionated heparin should be provided for ALI unless significant contraindications, serious bleeding within 48 hours, or recent surgery No high-quality data to suggest open vs. vascular intervention for COVID-associated ALI Coagulopathy, hyperinflammation, and endothelial injury increase morbidity post-vascular surgery Heparin resistance is common COVID-19 patients have abnormal coagulation patterns which may interfere with adequate therapeutic anticoagulation
Plastic Surgery	American Society of Plastic Surgeons (144)	Focuses mostly on transmissibility precautions Defers to CMS guidelines for resuming elective practice

• *Abbreviations: ACS, American College of Surgeons; ALI, acute limb ischemia; AV, arteriovenous; CMS, Centers for Medicare & Medicaid Services; COVID-19, coronarvirus disease 2019; ESAS, elective surgery acuity scale; ICU, intensive care unit; LEEP, loop electrosurgical excision procedure; LMWH, low molecular weight heparin; PCR, polymerase chain reaction; TVUS, transvaginal ultrasound.*

### Obstetrics

Obstetricians have long experience with surgical interventions on patients who have peripartum coagulopathies wherein the patient may switch between hemorrhagic and thrombotic phenotypes rapidly. Obstetricians have similar experience as CAC with pathologies such as preeclampsia, Hemolysis, Elevated Liver enzymes, and Low Platelets (HELLP) syndrome, morbidly adherent placenta (MAP) and other significant diseases associated with the peripartum state (145–156). This spectrum of disorders in the peripartum patient allow pathophysiological comparison to the hemostatic derangement of CAC in the nonpregnant surgical patient (29, 157). In each disease entity, there is endotheliitis that results in an immuno-thrombotic crosstalk resulting in local thrombosis and/or hemorrhage at the level of the inflamed endothelium (17, 157). For example, prepartum patients with preeclampsia must be given thromboprophylaxis to prevent clotting, but during parturition, they are at higher risk for bleeding, and again return to a hypercoagulable state postpartum (157). In tandem, postpartum patients with preeclampsia or other underlying risk factors for a hypercoagulable state should be treated for at least six weeks with thromboprophylaxis postpartum (146). Hence, discussion of the obstetrical approaches to providing thromboprophylaxis serves as an ideal foundation for describing the other surgical disciplines with more straightforward pathophysiological proclivities for the formation of perioperative clots. International guidelines for thromboprophylaxis for women who have undergone a C-section are heterogeneous in their recommendations regarding the timing and duration of thromboprophylaxis (146, 158).

In the United States and other countries, thromboprophylaxis for women undergoing C-sections has been described by the American College of Obstetricians & Gynaecologists guidelines that recommend thromboprophylaxis for all patients with C-sections who have at least one major indication or two minor indications for anticoagulation (159). The three major indications are previous VTE, body mass index (BMI) > 35, and thrombophilia. The minor indications are preeclampsia, hypertension, diabetes, peripartum infection, and other comorbidities (145, 159–163). Since guidelines are dynamic, the inclusion of COVID-19 as a minor indication seems reasonable. However, it would also be judicious to refer to the spectrum of diseases whereby women with severe COVID-19 are included in the group of patients with major indications for anticoagulation such that they would receive six weeks thromboprophylaxis. This paradigm has been useful in the most recent literature and serves as a template for applications to the other disciplines.

Since there is sparse literature regarding the guidance of thromboprophylaxis and treatment of the peripartum COVID-19 patient, surgeons must rely on consensus recommendations from interim guidelines. These recommendations for peripartum anticoagulation and thromboprophylaxis have been extrapolated from the COVID-19 literature for non-pregnant patients. Interim guidelines have been published by many organizations with specific references to pregnant women (164–166).

### Orthopaedics

Similar to obstetric patients, there is a spectrum of risk for developing postoperative VTE as a function of COVID-19 illness severity in patients undergoing orthopaedic surgery. Significant literature has demonstrated the benefit of serial VHAs to predict postoperative VTE (167–169). Therefore, for a disease which, by its very nature presents with a hypercoagulopathic state manifested clinically by increased VTEs and arterial thromboses, the use of VHAs in this COVID-19 population is a logical extension. While consensus recommendations have been proposed to optimize safety for patients undergoing elective surgery after previously being diagnosed with COVID-19, adjunctive VHAs may enhance these guidelines to better predict VTE and guide anticoagulant therapy postoperatively (131, 170–172).

Early intervention tends to be optimal for fracture fixation, but timing of surgery is historically complicated in patients with hemostatic derangement such as that which is induced in the post-COVID-19 patient. Decreased time between hip fracture and surgery correlates with decreased morbidity and mortality. However, correct timing of surgery in patients diagnosed with COVID-19 is difficult to assess (173). As an example of the heterogeneity of recommendations, it has been noted that a delay in fracture fixation of up to three months postinjury is acceptable, allowing for the risks of delayed surgery to be weighed against the risks of early fracture fixation performed during early post-COVID-19 prognosis (170).

Therefore, it is generally recommended that clinicians address each post-COVID-19 case individually and tailor a unique treatment plan for each patient based on their severity of injury and COVID-19 prognosis (172, 173). Patients waiting for elective orthopaedic surgery need to be reassessed in orthopaedic pathology, COVID-19 prognosis, surgical fitness, and desire to undergo the procedure with regards to evolving risks and benefits. Robust appraisal during the preoperative period to evaluate fitness for surgery is necessary to mitigate the risk of postoperative complications and decrease strain on postoperative intensive care units (170).

Specific orthopaedic society recommendations are outlined in **Table 2**. The American Association for Hand Surgery suggests delaying surgery based on the capacity to care for COVID-19 patients rather than postoperative prognosis (128). The American Orthopaedic Association offers broad suggestions regarding delay of surgery based on clinical need and institutional availability (130). Most of these orthopedic organizations do not have specific guidelines for delaying surgery to reduce the incidence of VTEs. However, the European Hip Society and European Knee Associates recommend delaying arthroplasty for six weeks following infection for otherwise healthy individuals, and for infected individuals with one or more comorbidities delaying the surgery for at least 8 weeks (131). When compared to anesthesia and non-orthopaedic society recommendations, the recommendations from European societies seem the most judicious provided the well-recognized increases in mortality and VTE complications associated with post-COVID-19 elective surgeries (13, 134). An example ESAS for delaying procedures is provided in **Table 3**.

**Table 3:** Orthopaedics Elective Surgery Acuity Scale (ESAS). All candidates for surgery should be assessed for surgical fitness on an individual basis. This is an example ESAS and not comprehensive. Procedure acuity for the individual patient may shift among tiers based upon acuity of illness, severity of COVID-19 infection and coagulopathy, and hospital surgical capacity (135). For specific length of operative delay, please see the text and Association of Anesthesiologists guidelines (13).

	Tier 1 Low Acuity Delay	Tier 2 Intermediate Acuity Delay if possible	Tier 3 High Acuity Do not delay
Trauma	n/a	Fractures >4 weeks old Chronic infections	All new fractures Acute infections Malunion/nonunion Patellar or quad tendon rupture
Orthopaedic Oncology	Benign tumor biopsy and removal	Aggressive benign tumor (e.g., giant cell tumor)	Pathologic fracture
Joints	Elective joint arthroplasty	Chronically infected hardware	Hip fracture or dislocation Acutely infected hardware

### General Surgery

The COVID-19 pandemic resulted in a significant reduction of general surgical procedures, including procedures for both benign and malignant diseases. While elective surgery for benign disease was intentionally halted during the pandemic, the reduction in procedures for malignancies was likely an unintentional consequence of reduced patient access to clinical examinations and diagnostic testing as a result of limited capacity of healthcare systems (174).

At the beginning of the pandemic, it was recommended that emergent cases be individualized based on patient risk, urgent cases be delayed at least until COVID-19 infection clears, and elective cases not be performed and potentially delayed for months (175). Emergency surgery was performed on patients regardless of their COVID-19 infection status.

The ACS and the American Society of General Surgeons initially published guidelines based on risk factors and patient benefits related to SARS-CoV-2 transmissibility during acute infection. These guidelines are heterogeneous and do not specifically address hemostatic complications related to elective and acute surgery during acute and convalescent COVID-19 (133, 134). These two associations adopted the CMS 3-tiered framework for urgency of procedures based on the ESAS (134, 136). This three-tiered system is defined by Tier 1, elective surgery which is to be postponed; Tier 2, intermediate acuity surgery postpone if possible; and Tier 3, indications for acute surgery where postponement is not an option (136). **Table 4** specifically defines example surgical procedures based on this three-tiered system.

**Table 4:** General Surgery Elective Surgery Acuity Scale (ESAS). All candidates for surgery should be assessed for surgical fitness on an individual basis. This is an example ESAS and not comprehensive. Procedure acuity for the individual patient may shift among tiers based upon acuity of illness, severity of COVID-19 infection and coagulopathy, and hospital surgical capacity (134–136). For specific length of operative delay, please see the text and Association of Anesthesiologists guidelines (13).

	Tier 1 Low Acuity Delay	Tier 2 Intermediate Acuity Delay if possible	Tier 3 High Acuity Do not delay
Abdominal/pelvic	Acute hemorrhoidal thrombosis/necrosis		Perianal or perirectal abscess Soft tissue infections Appendicitis^a^ Percutaneous cholecystectomy for severe acute cholecystitis Diverticulitis
Bariatric	Primary gastric bypass Gastric sleeve Gastric band Duodenal switch	Revisions for dysphagia Severe GERD Slipped band	Anastomotic leak Gastric perforation Obstruction
Breast	Excision of benign lesions Duct excisions Discordant biopsies likely to be benign Clinical stage T1N0 ER+/PR+/HER2- tumors	Any cancer that can receive neoadjuvant or hormonal therapy prior to surgery	Neoadjuvant patients finishing treatment Triple negative or HER2 + patients Excision of malignant recurrence Discordant biopsies likely to be malignant Clinical stage T2 or N1 ER+/PR+/HER2- tumors
Colorectal cancer	Prophylactic indications for hereditary conditions Large, benign appearing asymptomatic polyps Small, asymptomatic carcinoids	Locally advanced resectable colon cancer viable to neoadjuvant chemotherapy Rectal cancer with clear evidence of downstaging from neoadjuvant chemoradiation therapy	Perforated, obstructed, septic, or actively bleeding cancers Malignant polyps

*Abbreviations: ER, estrogen receptor; GERD, gastroesophageal reflux disease; HER2, human epidermal growth factor receptor 2; PR, progesterone receptor.*

^a^ *Evolving data suggests some careful selection for antibiotic treatment over surgery for uncomplicated appendicitis without appendicolith.*

With regards to transplant surgery, COVID-19 infection following organ implantation is associated with increased mortality (176, 177). Pre-vaccination prior to transplant has been suggested to reduce the risk of mortality (178). In the context of receiving an organ from a COVID-19 positive donor, there is growing evidence that viral transmission from abdominal organs is a nearly negligible risk (179). This has led to a call for increased use of COVID-19 positive donors without evidence of systemic disease, as the low risk of transmission does not appear to outweigh the potential lifesaving benefit from organ transplantation (180). However, a positive PCR test in a recipient should delay the procedure, and a delay of at least two to four weeks of negative serologies is required for waitlist reactivation of immunosuppressed candidates after COVID-19 contraction.

The Society of American Gastrointestinal Endoscopic Surgeons has provided specific guidelines for gastrointestinal cancers similar to the ESAS (138). A large study of over four million cancer patients found that most oncological procedures can be delayed by four weeks without a significant impact on patient morbidity or mortality (181, 182). However, a patient’s age and comorbidities are critical for weighing the relative benefit and risk of potentially exposing the patient to COVID-19 as opposed to choosing alternative options. The resources available to the surgeon must also be considered, since the pandemic has caused large fluctuations in patient acuity, volume, and hospital resources (183). In COVID-19 positive patients with primary or secondary cancer, elective liver or adrenal resection should be delayed until patients fully recover from acute COVID-19. In cases of jaundice or infection, percutaneous transhepatic biliary drainage (PTBD) or endoscopic retrograde cholangiopancreatography (ERCP) should be first implemented as a bridge therapy (184).

### Neurosurgery

The literature concerning timing of neurosurgical procedures has mostly considered SARS-CoV-2 transmissibility and healthcare worker safety (185–188). For example, it has been demonstrated that tracheostomies may be safely delayed two weeks, whereas craniectomy and tumor resections, as well as evacuation of subdural hematomas, need to be evaluated on a case-by-case basis (188, 189). Surgical treatment of chronic subdural hematoma, a usually successful intervention with a reported mortality rate of 3.7%, has a markedly increased mortality risk in COVID-19 positive surgical patients (190). Further investigation attributes multiple factors to this mortality rate, including thrombocytopenia, immune system impairment, and interstitial pneumonia progression post-surgery (191).

Like other surgical societies, there is heavy reliance on the modified ESAS for determining the timing of neurosurgical procedures for COVID (139). A similar 3-tiered system has been proposed regarding low, intermediate, and high acuity indications for neurosurgical intervention. Examples of surgeries that are divided into these three tiers are Tier 1, benign intracranial tumors which are low acuity; Tier 2, unruptured cerebral aneurysms and AV malformations as intermediate acuity; and Tier 3, malignant brain and spinal tumors for high acuity surgery where postponement is not an option (139). **Table 2 and 5** summarizes current neurosurgical guidelines regarding the timing of surgery for COVID-19 patients.

**Table 5:** Neurosurgery Elective Surgery Acuity Scale (ESAS). All candidates for surgery should be assessed for surgical fitness on an individual basis. This is an example ESAS and not comprehensive. Procedure acuity for the individual patient may shift among tiers based upon acuity of illness, severity of COVID-19 infection and coagulopathy, and hospital surgical capacity (135, 139). For specific length of operative delay, please see the text and Association of Anesthesiologists guidelines (13).

	Tier 1 Low Acuity Delay	Tier 2 Intermediate Acuity Delay if possible	Tier 3 High Acuity Do not delay
Neuro-oncology	Benign, asymptomatic intracranial tumors	Benign, symptomatic intracranial tumors	Malignant brain or spine tumors
Spine	Lumbar stenosis w/o FNDs Laminectomy discectomy w/o FNDs	Kyphoplasty Laminectomy discectomy w/ FND	Progressive cervical /thoracic myelopathy Cauda equina or conus medullaris syndrome
Neurovascular		AV malformation Unruptured aneurysm	Ruptured aneurysm coiling or clip Chronic subdural hematoma
Peripheral nerve	Carpal tunnel release	Peripheral nerve release	Brachial plexus injury
Other	Microvascular decompression of cranial nerves	DBS for Parkinson’s disease Refractory epilepsy	Post-traumatic elevated ICP not controlled by conservative measures

*Abbreviations: DBS, deep brain stimulation; FND, focal neurologic deficit; ICP, intracranial pressure.*

### Cardiothoracic and Vascular Surgery

Cardiothoracic and vascular surgery society guidelines have likewise adapted the CMS recommendations as well as ESAS guidelines for determining the timing of surgery during the COVID-19 pandemic (140, 141). The cardiothoracic guidelines have also benefited from the cardiology literature, which recommends adherence to standard anticoagulation procedures for patients with post-acute sequelae of COVID-19 who are undergoing percutaneous coronary intervention with or without angioplasty for ST segment elevation myocardial infarcts (192). During the acute period of COVID-19 infection, adherence to standard heparin boluses, anti-platelet, and intravenous and oral anti-factor agents is recommended without any change (193). For those patients whose intervention is complicated by a hypercoagulable state, treatment should be titrated with a personalized medicine approach and the use of VHAs to guide anticoagulation has been suggested (194–196). Acutely ill patients with severe coronary artery disease and valvular pathology not amenable to non-surgical interventions can either be operated immediately or temporized with a left ventricular assist device, intra-aortic balloon pump, or extracorporeal membrane oxygenation while waiting for the cytokine storm to abate if clinically possible (197). These guidelines are heterogeneous and likewise founded on the ASA and ACS three-tiered system of classifying patients with COVID-19 who require surgery (13, 134).

Provided the protracted nature of this pandemic, cardiac surgery programs must continue to proactively manage every patient on their waitlist with re-expansion of case volumes. Aspects of this management have been divided into three separate tiers of so-called ‘ramp up’ levels which determine the timing of surgery based on a combination of return to surgical case volume and acuity of indication for cardiothoracic surgery (141, 198).

The Canadian Cardiothoracic Society has described the following criteria: For ramp up phase one where the surgical volume is increased 0-25%, the patients who should avoid surgery when possible include frail elderly patients (clinical frailty score > 4) and vulnerable co-morbid patients including those with renal insufficiency, poor ejection fractions, and advanced congestive heart failure. Overall, in a phase one scenario, the cardiothoracic surgeon should attempt to avoid complex procedures including re-operative surgery while prioritizing CABG, isolated valve procedures, and less complex procedures to maximize patient flow. There should be an emphasis on patients with aortic stenosis and coronary artery disease with prognostic benefit, such as critical aortic stenosis and left main coronary disease. In the ramp up phase two, where surgical volume is increased 25-50%, inpatient urgent and emergency surgeries should be continued with broadening inclusion of appropriately prioritized outpatients. Ramp up phase three occurs with 50-100% increase in capacity with a return to normal outpatient services while continuing to prioritize those as greatest risk such as asymptomatic and severe mitral regurgitation, atrial septal defect or patent foramen ovale surgery, and asymptomatic aneurysm with demonstrated stable size.

In the thoracic surgery literature, a four-tier system of patient triage has been recommended by The Society of Thoracic Surgeons COVID-19 Task Force and the Workforce for Adult Cardiac and Vascular Surgery. For consistency here, we provide a three-tiered ESAS in **Table 6**.

**Table 6:** Cardiothoracic & Vascular Elective Surgery Acuity Scale (ESAS). All candidates for surgery should be assessed for surgical fitness on an individual basis. This is an example ESAS and not comprehensive. Procedure acuity for the individual patient may shift among tiers based upon acuity of illness, severity of COVID-19 infection and coagulopathy, and hospital surgical capacity (135, 140, 141). For specific length of operative delay, please see the text and Association of Anesthesiologists guidelines (13).

	Tier 1 Low Acuity Delay	Tier 2 Intermediate Acuity Delay if possible	Tier 3 High Acuity Do not delay
Aortic aneurysm	AAA < 6.5 cm	AAA or TAA > 6.5 cm	Ruptured or symptomatic AAA or TAA Associated with infection or prosthetic infection
Bypass graft complications	Asymptomatic bypass graft/stent stenosis	Revascularization for high grade re-stenosis of previous intervention	Infected arterial prosthesis
Carotid	Asymptomatic carotid artery stenosis		Symptomatic carotid stenosis: TCAR and CEA
Cardiac	Asymptomatic mitral regurgitation Isolated arrhythmia procedure	Symptomatic mitral regurgitation PFO or ASD surgery	Symptomatic aortic stenosis Severe CAD
Dialysis	n/a	Fistula revision for malfunction/ steal Fistulagram for malfunction AV fistula and graft placement for dialysis (ESRD, CK4, and CK5 only)	Infected dialysis access Tunneled dialysis catheters^a^ Thrombosed or nonfunctional dialysis access Fistula revision for ulceration
Mesenteric	n/a	Chronic mesenteric ischemia	Symptomatic acute mesenteric ischemia
Peripheral vascular disease	Surgical procedures for claudication	Chronic limb threatening ischemia	Acute limb ischemia
Thoracic outlet syndrome	Neurogenic TOS	TOS, venous	Symptomatic venous TOS with acute occlusion and marked edema TOS, arterial with thrombosis
Venous	Varicose veins IVC filter removal Asymptomatic May Thurner syndrome	Procedures for ulcerations secondary to venous disease IVC filter placement	Acute iliofemoral DVT with phlegmasia

*Abbreviations: AAA, abdominal aortic aneurysm; ASD, atrial septal defect; AV, arteriovenous; CAD, coronary artery disease; CEA, carotid endarterectomy; CK, chronic kidney disease; DVT, deep vein thrombosis; ESRD, end stage renal disease; IVC, inferior vena cava; PFO, patent foramen ovale; TAA, thoracic aortic aneurysm; TCAR, transcarotid artery revascularization; TOS, thoracic outlet syndrome.*

^a^ *May be placed in emergent situations which allow for postponement of the definitive fistula or graft surgery.*

Systemic anticoagulation with intravenous unfractionated heparin should be provided for all patients with acute limb ischemia (ALI). The performance of endovascular or open procedures for ALI is a function of the acuity of the limb ischemia and its relation to the hemostatic derangement present in the COVID-19 patient. There is no high-quality data to suggest that either open or vascular intervention is the preferred treatment ALI. It has been noted that a heparin resistance is in acute COVID-19 illness is common, while at the same time, the capricious nature of the cytokine storm causes the COVID-19 patient to have abnormal coagulation patterns. This interference with therapeutic anticoagulation requires frequent monitoring of not only aPTT and anti-Xa levels, but also with VHAs (17, 20–23, 108).

### Plastic, Oculoplastic, & Reconstructive Surgery

Similar guidelines based on the CMS and ESAS criteria have been adapted to the oculo/plastic and reconstructive procedures (199, 200). These guidelines are based on the three-tiered system which also incorporates the supply and demand for elective and acute surgeries and the acuity of the surgical intervention required. Of all specialties, the oculoplastic international societies have the most exhaustive list of procedures incorporated within the context of the three-tiered system since most of their surgeries are elective (201). These are summarized in **Table 7** below.

**Table 7:** Plastic, Oculoplastic, and Reconstructive Elective Surgery Acuity Scale (ESAS). All candidates for surgery should be assessed for surgical fitness on an individual basis. This is an example ESAS and not comprehensive. Procedure acuity for the individual patient may shift among tiers based upon acuity of illness, severity of COVID-19 infection and coagulopathy, and hospital surgical capacity (199–201). For specific length of operative delay, please see the text and Association of Anesthesiologists guidelines (13).

	Tier 1 Low Acuity Delay	Tier 2 Intermediate Acuity Delay if possible	Tier 3 High Acuity Do not delay
Oculoplastic	Blepharoplasty Slow-growing BCC Chalazion drainage Ptosis repair	Cutaneous malignancy other than slow-growing BCC Amblyopia in an infant Orbital exploration for non-vision threatening conditions	Canthotomy Orbital abscess drainage Optic nerve sheath fenestration Dacryocele decompression in a neonate
Plastic & Reconstructive	Revision reconstructive breast surgery Cosmetic surgery	Non-melanoma skin cancer biopsy/resection/grafting ORIF of maxillofacial fractures	Digit replantation Skull base reconstruction 3^rd^-degree burns Fasciotomy for acute compartment syndrome Necrotizing fasciitis debridement

*Abbreviations: BCC, basal cell carcinoma; ORIF, open reduction internal fixation.*

Of particular importance for this group of patients is the viability of microvascular flaps which, because of the hypercoagulability mediated by the immuno-thrombotic derangements that are part of CAC, require evaluation by the surgeon for the timing of surgery and the risk of ischemic flap loss. For example, criteria for the administration of continuous unfractionated heparin have been suggested for microvascular free flaps after head and neck cancer excision (202, 203). Recent publications have demonstrated the use of VHAs in guiding anticoagulation therapy to maintain vascular patency of microvascular vessels in flap surgery and reconstruction (204).

## Conclusion

The pathophysiological derangements of patients infected with SARS-CoV-2 require an understanding of the fundamental endotheliitis and immunothrombotic crosstalk causing fibrinolytic shutdown. For the surgeon who must confront an acutely ill patient with COVID-19, important decisions must be made regarding not just the timing of surgery, but also the nature of anticipating the thrombohemorrhagic complications of CAC and whether the patients are fully or prophylactically anticoagulated (205). For those patients who require elective surgery, a large study suggests a delay of up to seven weeks for elective surgery following SARS-CoV-2 infection, a guideline which has recently been adopted by specialty-specific societies (10). Yet, other studies have found abnormal hypercoagulability biomarkers up to six months after COVID-19 recovery, indicating potentially increased risk for some patients past seven weeks (81, 89–93). Refined use of these biomarkers for risk-benefit analysis can help to determine the most favorable time for surgery (7). In regard to the administration of thromboprophylaxis and full anticoagulation in the convalescent COVID-19 postoperative patient, certain biomarkers can potentially be used in a personalized-based medicine approach (77). More studies are needed to analyze biomarkers and physiologic derangements in COVID-19 recovery patients focusing on how these profiles forecast postoperative outcomes. VHAs have historically been used to profile postoperative patients at risk for VTE, and may help guide surgeons with timing of elective surgery, thromboprophylaxis, and full anticoagulation in this patient population (113–116). As the pandemic evolves, one can anticipate changing guidelines that may vary based on the COVID variant with accruing data on managing elective and acute surgery in this unique group of patients.

## Ethics Statement

Ethical clearance for microclot analysis was obtained from the Health Research Ethics Committee (HREC) of Stellenbosch University (South Africa): N19/03/043, project ID 9521. Confirmation of omicron variant: N20/04/008_COVID-19. Ethics approval for the inclusion of the microclot micrographs in this review paper was received from the Ethics committee of Stellenbosch University (HREC2-2022-24525).

## Author Contributions

Conceptualization, CMB, EEM, HBM, AVT, NZ, DF, WH, RM, BSB, and MMW; Writing – Original Draft Preparation, CMB, AVT, NZ, JZ, SZ, TJB, MB, HB, RM, BSB, and MMW; Writing – Review & Editing, CMB, EEM, HBM, MDN, AVT, NZ, JZ, SZ, TJB, MB, HB, GW, DF, WH, HCK, GL, GJL, PJL, EP, MJK, MSM, SS, TGM, DBK, MDF, LG, RZK, CNM, RM, RM, BSB, and MMW; Visualization, AVT, MMW; Supervision, AVT, MMW; Project Administration, CMB, AVT, NZ, and MMW; Funding Acquisition, MMW, EP. Patient recruitment, fluorescent microscopic and viral sequencing analysis, GJL, PJL, EP, MJK, MSM, SS, TGM, DBK. All authors contributed to the article and approved the submitted version.

## Funding

DBK: Novo Nordisk Foundation for support (grant NNF20CC0035580) (Project Code 96825). MJK and TGM: Research reported this article was supported by the South African Medical Research Council with funds received from the Department of Science and Innovation (Project Code 96825). The content and findings reported and illustrated are the sole deduction, view and responsibility of the researchers and do not reflect the official position and sentiments of the funders.

## Conflict of Interest

EEM: Research support from Haemonetics, Instrumentation Laboratory, Hemosonics, Stago, and Diapharma. HBM: Research support from Haemonetics and Instumentation Laboratory. MJK is a non-executive director and shareholder of Gknowmix (Pty) Ltd. EP is the managing director of BioCODE Technologies. MMW is on the speaker’s bureau for AstraZeneca.
